# Level of Anxiety Among Healthcare Workers at a Comprehensive Cancer Center During the Coronavirus Disease 2019 Pandemic

**DOI:** 10.7759/cureus.18089

**Published:** 2021-09-19

**Authors:** Hosam A Alghanmi, Emad E Tashkandi, Doaa K. Mohorjy, Abdullah M. Alqahtani

**Affiliations:** 1 Department of Medical Oncology, Oncology Center, King Abdullah Medical City, Makkah, SAU; 2 Research Center – Science and Technology Unit, King Abdullah Medical City, Makkah, SAU; 3 Department of Mental Health, Neuroscience Center, King Abdullah Medical City, Makkah, SAU

**Keywords:** covid-19, medical oncology staff, anxiety level, pandemics, healthcare

## Abstract

Objective

The recent coronavirus pandemic (SARS-CoV-2) has severely increased the burden on the field of oncology, leading to the implementation of new rules to overcome its negative impact. An important issue is the psychological impact of the pandemic on already vulnerable populations, such as oncology staff, as reported by oncology associations.

This study assessed the anxiety level among oncology staff during the coronavirus disease 2019 pandemic and related it to its risk factors and the effectiveness of departmental interventions that seek to provide coping methods.

Methods

A cross-sectional survey was administered to the oncology staff at our oncology center. Generalized anxiety disorder scale “yes-or-no” questions were used to determine secondary objectives regarding the concern of infection and the impact of a departmental intervention on overcoming distress.

Results

Overall, 199 participants were included in the analysis; more than 60% were women, with a median age of 34 years. More than 50% had anxiety and the most significant risk factors were parenthood and contraction of infection (P-value 0.03 and 0.02, respectively). Most responders reported an increase in the workload during the pandemic, but coping methods applied by the administration had improved anxiety levels as reported by a majority of the responders.

Conclusion

Oncology staff members have been negatively affected by the pandemic, although coping methods helped to improve anxiety. In the future, attention must be focused on the most vulnerable groups.

## Introduction

A novel type of pneumonia was reported in Wuhan at the end of December 2019 [1]. On February 11, 2020, the World Health Organization (WHO) named the novel coronavirus disease - coronavirus disease 2019 (COVID-19) [2]. Severe acute respiratory syndrome coronavirus 2 (SARS-CoV-2), the causative agent of the disease, belongs to a large family of ribonucleic acid viruses. It causes symptoms that range from the common cold to those similar to the Middle East respiratory syndrome (MERS) and acute respiratory syndrome (SARS) [3]. After the announcement of COVID-19 as a pandemic, fear and anxiety started escalating in the general population and healthcare workers. Psychological drawbacks among healthcare workers have been reported during previous pandemics such as Ebola virus disease and MERS [4-5]. A wide range of psychological disorders has been reported among healthcare workers, including anxiety, insomnia, depression, burnout, and post-traumatic disorder [6]. Extreme situations can provoke suicide, as reported in 2 countries [7-8].

Many factors may increase the propensity of healthcare workers to psychological stress such as young age, female sex, contact with infected patients, psychiatric background, and fear of spreading the infection to family members [9]. Other contributing factors to the drawbacks of a pandemic are the lack of protective personal equipment, widespread media coverage, lack of therapy, and increased workload [10]. Among healthcare workers, the psychological impact was greater in the nursing staff, particularly the intensive care and emergency unit staff [10]. According to a narrative review on the impact of COVID-19 on mental health, the authors found anxiety to be the most common mental health symptom [11]. A study in Saudi Arabia conducted during the COVID-19 pandemic among healthcare providers reported the prevalence of anxiety and depression to be 51.4 % and 55.2%, respectively [1]. In response to the COVID-19 pandemic, the State Council of the People’s Republic of China announced the implementation of psychological assistance services through telephone, internet, and other services to help decrease anxiety levels [10]. Additionally, many programs have been implemented to enhance best practice measures in addition to training. Healthcare workers at our cancer center still assist suspected or confirmed cases of COVID-19 because it is a referral center for different regions. Furthermore, patients with cancer are immunocompromised and more prone to infection and morbidity due to COVID-19 than other patients [12]. Therefore, we decided to focus our study on healthcare workers of a cancer center, as they have been reported to have more psychological stress as compared to healthcare workers of other specialties and, hence, are more troubled by this pandemic [13]. Initially, a report from the European Society for Medical Oncology demonstrated that 71% of young oncologists experienced burnout and 45% experienced emotional exhaustion [14]. Another report released by the American Society of Clinical Oncology, which evaluated the result of stress among the subscribers of the journal, found that more than half of the participants had burnout [15].

The impact of pandemics not only affects the oncology staff but also extends to patients with cancer and their caregivers. A study in Singapore conducted among oncology staff, caregivers of patients with cancer, and patients reported that more than half of the patients and caregivers had high levels of fear of COVID-19 and less than half of healthcare workers reported the same experience. In the same study, the prevalence of anxiety among healthcare workers was higher among those who had burnout [16]. This is the first study to investigate the psychological impact of the COVID-19 pandemic on an oncology staff in the Kingdom of Saudi Arabia. The aim of this study was to evaluate the level of anxiety during the COVID-19 pandemic among the healthcare providers of a cancer center using the Generalized Anxiety Disorder (GAD) score, a validated score, to screen for anxiety [17]. Additionally, we identified predisposing and risk factors associated with the development of different levels of anxiety in our study population and assessed the success of proposed departmental interventions that may help overcome the drawbacks of the pandemic.

## Materials and methods

Study design

A cross-sectional observational study (survey) was conducted among the healthcare workers of King Abdullah Medical City in Makkah.

Study population

A list of all healthcare workers at our cancer center was obtained from the cancer center administration. Individuals who met the inclusion criteria were requested to complete the survey as a web-based questionnaire distributed by the investigators.

Ethical consent and confidentiality

This study was approved by the Institutional Review Board of King Abdullah Medical City. Survey responses were collected anonymously. No identified identifiable or private information was collected, and all responses were kept confidential. This study was carried out in accordance with the Declaration of Helsinki.

Inclusion and exclusion criteria

This study included medical oncology, hemato-oncology, palliative medicine, radiation oncology, and allied health caregivers (pharmacists, nurses, social workers, physiotherapists, occupational therapists) managing patients with cancer (either inpatients or outpatients) during the COVID-19 pandemic. Health staff who were not working with cancer patients or who were working at other cancer centers were excluded from the study.

Study procedure and materials

Our survey was generated using the Generalized Anxiety Disorder (GAD) scale. The interview-based questionnaires comprised 20 items that focused on three areas: characteristics of healthcare workers, COVID-19 pandemic exposures, and anxiety level assessment. The survey used “Yes or No” questions that tested for departmental intervention during the pandemic.

A convenient sample design was used. To identify our target population, the cancer center administration was approached to conduct the interview for the healthcare workers. Email and WhatsApp were used to set up a suitable time for meeting and conducting the survey. Five experts in oncology, psychiatry, and methodology were invited to review and modify the survey. After the nomination of items, they were ranked to achieve a consensus on the nominated items. The methodology and content experts performed a subsequent review to eliminate redundant items using binary responses (excluding and including).

Statistical analysis

Data analysis was performed using SPSS Statistics software (version 25; IBM Corp., Armonk, NY). For categorical variables, counts and percentages were used, while continuous and ordinal variables were expressed as mean ± standard deviation. The student’s t-test was used to determine the association between continuous variables, and Pearson’s chi-square test (or Fisher’s exact test, where appropriate) was used to evaluate categorical variables. The significance level for all tests was set to α = 0.05, and all tests were two-tailed.

## Results

Among the 227 healthcare workers, 199 were included. The cohort included 126 women (63.3%) and 73 men (36.7%). The mean age was 35.44 years. Overall, 156 participants (78.4%) were married, 93 (46.7%) were doctors, 142 (71.4%) had children, and 95 (47.7%) worked with inpatients. Diabetes was observed in 17 participants (8.5%), hypertension in 21 (10.6%), ischemic heart disease in 6 (3%), and psychiatric disorders in 7 (3.5%).

COVID-19 was confirmed in 35 participants (17.6%) and suspected in 70 (35.2%) (Table 1). Additionally, 46 participants (23.1%) had a family member with COVID-19 and 117 (58.8%) were in contact with a confirmed patient. Moreover, 152 (76.4%) had experienced an increase in workload during the pandemic. Table 2 lists the responses to the items related to anxiety in relation to COVID-19. Table 3 depicts the responses to questions related to SARS-CoV-2 infection. Comparisons of the staff’s demographic data and anxiety scale showed that having children (p=0.036) and having been a confirmed case of COVID-19 (p=0.024) were significantly related to a high anxiety level (Table 4).

**Table 1:** Staff demographics and patients’ COVID-19-related comorbidities SD: standard deviation

Variables	Overall (n=199)
Demographics	
Sex:	
Male	73 (36.7%)
Female	126 (63.3%)
Age (in years):	
Mean ± SD	35.44 ± 6.43
Median (Min–Max)	34 (24–55)
Marital status:	
Single	41 (20.6%)
Married	156 (78.4%)
Widowed	1 (0.5%)
Prefer not to answer	1 (0.5%)
Children:	
Yes	142 (71.4%)
Profession:	
Doctor	93 (46.7%)
Pharmacist	17 (8.5%)
Nurse	67 (33.7%)
Social worker	5 (2.5%)
Physiotherapist	8 (4%)
Patient relation	8 (4%)
Ward clerk	1 (0.5%)
Location of work:	
Outpatient	57 (28.6%)
Inpatient	95 (47.7%)
Both	47 (23.6%)
Comorbidity:	
Diabetes	17 (8.5%)
Hypertension	21 (10.6%)
Ischemic heart disease	6 (3%)
Psychiatric background	7 (3.5%)

**Table 2:** Anxiety scale assessment St: statement

St	Items	% of responses (N= 199)
1	Nonexistent	89 (44.7%)
2	Mild (6–10)	63 (31.7%)
3	Moderate (11–15)	27 (13.6%)
4	Severe (16–21)	20 (10.1%)

**Table 3:** COVID-19-related questions

Were you a confirmed case of COVID-19? Yes	35 (17.6%)
Were you suspected of having COVID-19? Yes	70 (35.2%)
Did you think that the workload increased during COVID-19? Yes	152 (76.4%)
Was anyone in your family affected? Yes	46 (23.1%)
Have you come in contact with a confirmed patient? Yes	117 (58.8%)

**Table 4:** Anxiety scale among the different demographic groups St: statement

St	Items	Nonexistent (0–5)	Mild (6–10)	Moderate (11–15)	Severe (16–21)	P-value
1	Sex					0.128
	Male	39 (19.6%)	18 (9%)	7 (3.5%)	9 (4.5%)	
	Female	50 (25.1%)	45 (22.6%)	20 (10.1%)	11 (5.5%)	
2	Profession					0.554
	Doctor	40 (20.1%)	28 (14.1%)	11 (5.5%)	14 (7%)	
	Pharmacist	9 (4.5%)	5 (2.5%)	2 (1%)	1 (0.5%)	
	Nurse	31 (15.6%)	20 (10.1%)	12 (6%)	4 (2%)	
	Allied health professional	9 (4.5%)	10 (5%)	2 (1%)	1 (0.5%)	
3	Marital status					0.095
	Single	19 (9.5%)	11 (5.5%)	11 (5.5%)	0	
	Married	68 (34.2%)	52 (26.1%)	16 (8%)	20 (10.1%)	
	Widowed	1 (0.5%)	0	0	0	
	Prefer not to answer	1 (0.5%)	0	0	0	
4	Children					0.036
	Yes	64 (32.2%)	46 (23.1%)	14 (7%)	18 (9%)	
	No	25 (12.6%)	17 (8.5%)	13 (6.5%)	2 (1%)	
5	Confirmed COVID-19					0.024
	Yes	8 (4%)	17 (8.6%)	5 (2.5%)	5 (2.5%)	
	No	81 (40.9%)	45 (22.7%)	22 (11.1%)	15 (7.6%)	
6	Suspected of COVID-19					0.090
	Yes	23 (11.6%)	17 (13.6%)	11 (5.6%)	9 (4.5%)	
	No	66 (33.3%)	35 (17.7%)	16 (8.1%)	11 (5.6%)	

Table 5 shows the departmental interventions used to cope with the pandemic distress of the study participants, summarized as frequencies and percentages. Based on the evaluation of the open-ended questions, postponing the visit of patients with symptoms of common cold and increasing the number of staff members were considered appropriate measures by the hospital staff. With respect to staff support, the implementation of an easily accessible psychological support system and financial compensation were the suggested measures.

**Table 5:** Departmental interventions to cope with the pandemic distress St: Statement

St.	Items	No. of responses (%) (N=199)
Making the duty one week on and the other week off, virtual clinic, virtual contact with the patient’s relatives		
1	Do you think it helps to decrease the stress level? Yes	162 (81.4%)
2	Do you think it makes workload more acceptable? Yes	151 (75.9%)
3	Do you think it gives time to relax and be ready for a heavy workload? Yes	162 (81.4%)
4	Do you think departmental intervention on the implementation of telemedicine can help decrease the stress level? Yes	147 (73.9%)
Psychological support before or during the pandemic		
1	Have you received any psychological support during the pandemic? Yes	52 (26.1%)
2	Do you think the implementation of psychological support at the beginning of a pandemic would help to cope with its impact? Yes	166 (83.4%)

## Discussion

With the growing body of evidence regarding the impact of pandemics on the mental health or psychological state of healthcare workers, some hospitals have initiated psychological interventions and psychiatric screening programs to overcome these concerns. In our study, the majority of healthcare practitioners were women with a median age of 34 years. The majority of healthcare staff was married and had children. They were mainly doctors, and the majority suffered anxiety (mild, 31.7%; moderate, 13.6%; and severe, 10.1%). The strongest factors associated with increased anxiety were parenthood and being confirmed of having COVID-19. Less than a third of the participants received psychological support, and more than two-thirds agreed on having increased workload during the pandemic and on departmental interventions being helpful in overcoming stress.

Our study showed that the majority of the oncology staff suffers from anxiety. Compared with other studies conducted on oncology staff of two pandemic-affected regions, the frequency of anxiety in our staff was higher. A study conducted in Singapore showed that 14% of the medical staff (including doctors, nurses, and cancer caregivers) reported anxiety, whereas, in France, the frequency of anxiety was 32% [16,18]. Another study conducted on oncology physicians in the United States of America reported a prevalence of anxiety and depression of 62% and 23%, respectively, with demographic factors such as female gender, younger age, and fewer years of experience being associated with greater anxiety [19]. Burnout is another negative impact of pandemics that was detected in Chinese oncology staff during the COVID-19 pandemic; its incidence was higher in oncology staff working in the COVID-19 ward (15%) than in those working in other wards [20]. Although our results clearly showed the negative psychological aspects of the current pandemic in our staff, we investigated only one of the adverse consequences of the pandemic: anxiety. In the French and Singaporean studies, other negative aspects such as depression, fear, and burnout were assessed.

Despite the high percentage of staff experiencing anxiety observed in our study, only 26% sought psychological advice. This could be due to a lack of awareness regarding the need to obtain psychological support. The proportion of the staff that had obtained psychological assistance was very low, as occurred in the French study, where only 4% sought assistance [18]. In this regard, a Chinese study reported that medical staff are less likely to ask for psychological assistance than people from other professions [21].

The concern of transmitting the infection to family members is a predisposing factor for negative psychological impact [9,22]. In contrast, a study has suggested that being married and older are protective factors against negative impacts, as well as having a family during the quarantine [23]; however, it is worth noting that this study was conducted in the general population, not in healthcare workers. The nursing staff is the most vulnerable group, being at risk of more negative impacts from the pandemic, as reported in previous studies on pandemics [10,24]. Their direct contact with patients for prolonged periods may help explain this result. Our study showed that the level of mild, moderate, and severe anxiety among nurses was 10%, 6%, and 2%, respectively. In our study, nurses were not the most affected group, maybe due to the lower number of nurse participants. Substantial reports from previous studies agree that nurses have a higher prevalence of negative consequences during pandemics.

Additionally, according to our findings, pharmacists had lower levels of anxiety than the other professions (mild, 2.5%; moderate, 1%; and severe, 0.5%). This could be because of the small number of pharmacist participants and the implementation of drug delivery services during the COVID-19 pandemic for patients on oral treatment. Another study reported that the mean anxiety score among pharmacists was 6.98-5.01 as compared with 6.52-4.38 in physicians and 7.23-4.27 in nurses [1]. Hospital pharmacists are frontline healthcare workers and are directly involved in patient care with physicians and nurses [25]. The mental health of pharmacists was one of the top four concerns during the COVID-19 pandemic according to the Canadian Pharmacist Association [26]. Further studies are required to investigate the negative impact of the pandemic on pharmacists.

Although the allied services staff, such as patient relations, social workers, and physiotherapists, accounted for a small percentage of the study population (11%), a lower anxiety level was observed among them in comparison with other staff members (5%, 1%, 0.5%; mild, moderate, and severe). A study from China during the H1N1 influenza pandemic that included allied services staff found that 1.7% of them experienced severe anxiety, a lower rate than that of the medical staff [22]. In our study, this may be explained by their limited contact with patients and the use of virtual technology.

Our study confirmed that women had a higher prevalence of moderate and severe anxiety than men (10.1% versus 3.5%; and 5.5% versus 4.4%, respectively) than men. Likewise, when considering particular row totals, 8.7% of women experienced high levels of anxiety, compared to 12.3% of men. Additionally, more women (35%) than men (25%) seemed to perceive low levels of anxiety, suggesting that, although they are aware of the situation, women may have better coping mechanisms to avoid reaching higher levels of anxiety. This finding is consistent with a study from China during the COVID-19 pandemic reporting the percentage of women suffering from anxiety (34%, 66.7%, and 5.8% for mild, moderate, and severe, respectively) to be higher than that of men [10]. Our findings demonstrated a higher prevalence of anxiety in parents compared with single or married individuals with no children; this applied for all anxiety levels, with a significant difference in severe anxiety (9% vs. 1%, respectively). These findings are supported by studies from previous pandemics and different countries. Although the percentage of infected participants in our study was 17%, and that of suspected cases was 35%, the level of anxiety was not higher in these groups. This could be because suspected or confirmed cases of SARS-CoV-2 infection are isolated in their homes and thus have more time for relaxation, or because being infected gives them active immunity against the virus, reducing the risk of morbidity from persistent exposure. The aforementioned aspects must be considered during pandemics to focus efforts on the most vulnerable groups. Consequently, coping interventions from the administration are required in the newly emerging pandemic to overcome its negative impact [21]. Coping methods that help decrease psychological impact at our institute are the implementation of a new duty schedule that includes alternating between one week on and one week off, virtual clinics, and virtual contact with the patient’s relatives, as opposed to visiting the hospital. This was agreed to decrease stress by 73% of participants. The majority (80%) of participants reported that psychological support, if initiated at the beginning of a pandemic, would help in coping with anxiety. Coping measures include receiving support from the manager, talking to someone about concerns, having religious beliefs, receiving support from colleagues, and receiving psychological assistance [21]. A study from China reported that methods to decrease the negative impact of the pandemic through motivation include strict infection control and recognition of the staff’s efforts [10].

At the end of the survey, we listed open questions for final suggestions to improve the experience of the current and subsequent pandemics. A total of 47 suggestions were divided into two categories: staffing and support. Suggestions related to staffing included postponing elective cases, allowing internal vacation, increasing the staff number, conducting workshops on how to deal with pandemics, and decreasing working hours. Support-related suggestions emphasized financial compensations and the implementation of easy access to psychological support. Although some of the suggestions were considered, they required approval from a higher authority.

Strengths

This study focused on the most common psychological consequence of a pandemic: anxiety. Additionally, it involved a specific group of healthcare workers that is more likely to experience negative psychological impacts, even in the absence of pandemics.

Limitations

This study had some limitations. First, the findings from our cross-sectional and single-center research are not generalizable. Second, we investigated the impact of the pandemic exclusively on anxiety levels. However, this aspect will continue to be affected after the pandemic, which opens opportunities for future studies in the post-pandemic era.

Implications

Our findings are in line with those of most studies exploring the negative impact of pandemics. The implementation of coping methods to decrease pandemic distress by focusing on vulnerable groups, considering early psychological intervention and support, will be useful to improve mental health among health workers.

Future plan

The implementation of systems to spread awareness and psychological support for the most vulnerable groups and frontline healthcare workers at the beginning of any pandemic is required. The drawbacks of the pandemic remain even after its end, and more studies should be conducted on post-traumatic disorders.

## Conclusions

The psychological impact of pandemics has recently raised concerns. The current study findings have highlighted concerns regarding the difficulties encountered during the pandemic by oncology staff members who already experience distress owing to the specialty itself. Hence, psychological support, including the early implementation of coping methods, should be readily available for people, particularly those affected by the pandemic. On the whole, more research on the coping methods needed to overcome the negative impact of pandemics among health care workers is required, and further studies should be conducted at the end of pandemics to assess the efficacy of the coping methods and the post-pandemic negative impacts.
